# Characterization of pulmonary impairment associated with COVID-19 in patients requiring mechanical ventilation

**DOI:** 10.5935/0103-507X.20210007

**Published:** 2021

**Authors:** Edouard Virot, Cyrille Mathien, Valentin Pointurier, Antoine Poidevin, Guylaine Labro, Luis Pinto, Louise Marie Jandeaux, Joy Mootien, Khaldoun Kuteifan

**Affiliations:** 1 Medical Intensive Care Unit, Hôpital Emile Muller, Groupe Hospitalier de la Région de Mulhouse et Sud-Alsace - Alsace - Mulhouse, France.; 2 Antibiotic Stewardship Unit, Hôpital Emile Muller, Groupe Hospitalier de la Région de Mulhouse et Sud-Alsace - Alsace - Mulhouse, France.

**Keywords:** SARS-CoV-2, COVID-19, Coronavirus infections, Acute respiratory distress syndrome, Thermodilution, Pulmonary edema, SARS-CoV-2, COVID-19, Infecções por coronavírus, Síndrome de dificuldade respiratória, Termodiluição, Edema pulmonar

## Abstract

**Objective:**

To detect early respiratory and hemodynamic instability to characterize pulmonary impairment in patients with severe COVID-19.

**Methods:**

We retrospectively analyzed data collected from COVID-19 patients suffering from acute respiratory failure requiring intubation and mechanical ventilation. We used transpulmonary thermodilution assessment with a PiCCO™ device. We collected demographic, respiratory, hemodynamic and echocardiographic data within the first 48 hours after admission. Descriptive statistics were used to summarize the data.

**Results:**

Fifty-three patients with severe COVID-19 were admitted between March 22nd and April 7th. Twelve of them (22.6%) were monitored with a PiCCO™ device. Upon admission, the global-end diastolic volume indexed was normal (mean 738.8mL ± 209.2) and moderately increased at H48 (879mL ± 179), and the cardiac index was subnormal (2.84 ± 0.65). All patients showed extravascular lung water over 8mL/kg on admission (17.9 ± 8.9). We did not identify any argument for cardiogenic failure.

**Conclusion:**

In the case of severe COVID-19 pneumonia, hemodynamic and respiratory presentation is consistent with pulmonary edema without evidence of cardiogenic origin, favoring the diagnosis of acute respiratory distress syndrome.

## Abstract (pt)

**Objetivo:**

Detectar precocemente a instabilidade respiratória e hemodinâmica para caracterizar o comprometimento pulmonar em pacientes com COVID-19 grave.

**Métodos:**

Analisamos retrospectivamente os dados colhidos de pacientes com COVID-19 que apresentaram insuficiência respiratória aguda com necessidade de intubação e ventilação mecânica. Utilizamos a avaliação da termodiluição transpulmonar por meio do dispositivo PiCCO™. Foram coletados os dados demográficos, respiratórios, hemodinâmicos e ecocardiográficos dentro das primeiras 48 horas após a admissão. Para resumir os dados, utilizamos estatística descritiva.

**Resultados:**

Entre 22 de março e 7 de abril de 2020, foram admitidos 23 pacientes com COVID-19 grave. Foram monitorados com o dispositivo PiCCO™ 12 (22,6%) deles. Quando da admissão, o volume diastólico final global indexado era normal (média de 738,8mL ± 209,2) e, na hora 48, encontrava-se moderadamente aumentado (879mL ± 179), enquanto o índice cardíaco se achava abaixo do normal (2,84 ± 0,65). Todos os pacientes revelaram a presença de água extravascular pulmonar acima de 8mL/kg na admissão (17,9 ± 8,9). Não identificamos qualquer evidência de origem cardiogênica.

**Conclusão:**

No caso de pneumonia grave por COVID-19, o quadro hemodinâmico e respiratório é compatível com edema pulmonar sem evidência de origem cardiogênica, o que favorece o diagnóstico de síndrome do desconforto respiratório agudo.

## INTRODUCTION

A novel coronavirus, the severe acute respiratory syndrome coronavirus 2 (SARS-CoV-2), causing respiratory infections (coronavirus disease 2019 - COVID-19), was first detected in Wuhan, China, in December 2019.^(1-3)^ The virus spread around the world within a few weeks.^(4)^ The first three cases of COVID-19 identified in France were imported cases and were confirmed on January 24^th^, 2020, in persons who had recently visited Wuhan. Quickly, the virus spread around France initially among localized clusters.^(5)^ An important cluster was identified in the city of Mulhouse, Alsace, France, after a religious meeting with a gathering of approximately 2000 people held from the 17^th^ to 24^th^ of February 2020.^(6)^ SARS-CoV-2 can cause severe pneumonia, and some patients require mechanical ventilation.^(7,8)^ As of March 3rd, more than 400 patients with severe pneumonia requiring mechanical ventilation were admitted to the Mulhouse public hospital. The pathophysiology of severe respiratory failure remains unclear.^(9)^ To optimize treatment and better understand the pathophysiology of respiratory impairment in patients with severe COVID-19 pneumonia, we used transpulmonary thermodilution with a PiCCO™ device (Pulsion Medical Systems, Munich, Germany).

This study aims to report the early hemodynamic and respiratory instability of these patients.

## METHODS

We retrospectively analyzed data collected from COVID-19 patients suffering acute respiratory failure with severe hypoxemia admitted to the medical intensive care unit (ICU) at the Mulhouse Hospital, France. All patients were sedated and underwent assisted-controlled mechanical ventilation prior to transpulmonary thermodilution assessment with a PiCCO™ device. A PiCCO™ catheter was arbitrarily inserted when one of our three monitors was available: 2 PiCCO2™ and 1 PiCCO Plus™ (Pulsion Medical Systems, Munich, Germany). The injection site of the boluses of cold saline was exclusively the superior vena cava territory. An arterial PiCCO™ catheter was inserted in either a radial or femoral site.

The following data were collected from records: demographic data, Sequential Organ Failure Assessment (SOFA), Simplified Acute Physiology Score II (SAPS II) and associated comorbidities. Other data were collected from medical records completed every four hours during the ICU stay. Respiratory data included oxygen saturation (SpO_2_), respiratory rate, tidal volume, positive end expiratory pressure (PEEP), and static compliance of the respiratory system. Hemodynamic data included systolic arterial blood pressure, diastolic arterial blood pressure, mean arterial blood pressure and temperature. Transpulmonary thermodilution parameters included cardiac index, global end diastolic volume (GEDV) indexed to body surface area, extravascular lung water indexed to the ideal body weight (EVLW) and systemic vascular resistance index. Transpulmonary thermodilution assessment was performed every six hours, and parameters were collected at H0, H6, H12, H18, H24, H36 and H48.

Echocardiographic parameters were collected when the exam was performed within 48 hours following the first transpulmonary thermodilution. Left ventricular pressure was assessed by the ratio of mitral inflow E wave velocity to early diastolic mitral annulus velocity (E/E’) measured by tissue Doppler imaging. The inferior vena cava diameter and subaortic velocity time integral were also evaluated. Catecholamine doses and fluid load between every transpulmonary thermodilution were also collected. Biological parameters and the partial pressure of arterial oxygen to fraction of inspired oxygen (PaO_2_/FiO_2_) ratio were collected on a daily basis.

### Statistical analysis

Descriptive statistics were used to summarize the data. Descriptions of quantitative variables are reported as medians and interquartile ranges or means and standard deviations, as appropriate. Categorical variables are summarized as counts and percentages. Analysis was performed with R software version 3.5.2.

The study was approved by a local ethics committee named *Comité d’Ethique du Groupe Hospitalier de la Région de Mulhouse et Sud-Alsace*. Every patient and/or family was informed that anonymous data could be used for research.

## RESULTS

**Figure 1:**
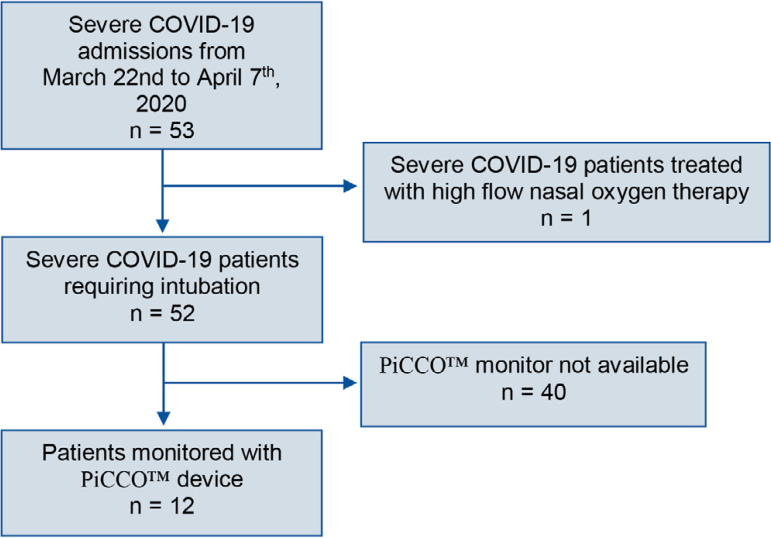
Study participants.

Fifty-three patients with severe COVID-19 pneumonia were admitted to our ICU from March 22^nd^ to April 7^th^. Confirmed COVID-19 was defined by a positive result on a reverse transcriptase polymerase chain reaction (RT-PCR) test from nasopharyngeal swabs. All of the patients were admitted with acute respiratory failure secondary to severe COVID-19 pneumonia. Chest radiography was performed for every patient upon ICU admission and was consistent with extensive bilateral pulmonary opacities. Only 1 patient did not require intubation and was treated with high-flow nasal oxygen therapy. Among the other 52 ventilated patients, 12 were monitored with the PiCCO™ device. The flow chart of this study is reported in figure 1. The first measure by transpulmonary thermodilution was obtained within 6 hours after intubation. There was a predominant number of males (n = 9; 75%). The mean age was 53.4 years, and the majority had no comorbidities. The different characteristics of these patients are reported in table 1. One patient presented limb ischemia 18 hours after PiCCO™ catheter insertion. It was resolved after catheter extraction without surgical intervention. We did not observe any other complications due to PiCCO™ monitoring.

**Table 1:** Demographic characteristics of the 12 patients monitored with the PiCCO^™^ device

Patients	
Age	53.4 ± 21.8
Sex male	9 (75)
SAPS II	48.8 ± 17.7
SOFA	7.7 ± 2.9
Body mass index	33.6 ± 7.6
Current smoker	0
Hypertension	5 (41.7)
Diabetes mellitus	1 (8.3)
Chronic respiratory failure	1 (8.3)
Chronic heart failure	1 (8.3)

SAPS II - Simplified Acute Physiology Score II; SOFA - Sequential Organ Failure Assessment. Results expressed as the mean ± standard deviation or n (%).

### Hemodynamic features

On admission, the mean arterial blood pressure was 79.9mmHg (± 12.6). The mean heart rate was 96.5bpm (± 20). Only two patients required norepinephrine > 0.35µg/kg/minute. The mean dose of norepinephrine was low (0.26µg/kg/minute ± 0.37). Three patients received a briefly low dose of dobutamine (2 to 5µg/kg/minute). Nine patients (75%) underwent echocardiographic evaluation within the first 48 hours. None of the patients had elevated left ventricular pressure assessed by E/E’ (mean 5.6 ± 1.4). The inferior vena cava diameter was elevated (mean 20.1 ± 2.4). The hemodynamic characteristics are reported in table 2.

**Table 2:** Hemodynamic characteristics of patients monitored with PiCCO^™^ device

	H0 (n = 12)	H24 (n = 11)	H48 (n = 11)
Systolic blood pressure (mmHg)	116 (19)	121 (17)	114 (9)
Diastolic blood pressure (mmHg)	62 (11)	61 (8)	59 (11)
Mean blood pressure (mmHg)	80 (13)	81 (10)	77(9)
Heart rate (bpm)	96 (20)	81 (15)	79 (11)
Epinephrine (*µ*g/kg/minute)	0.21 (0.07 - 0.21)	0.12 (0 - 0.28)	0.08 (0.03 - 0.19)
Dobutamine (*µ*g/kg/minute)	0	0.18 (0-5)	0
PEEP (mmHg)	13.2 ± 1.6	12.7 ± 1.9	13 ± 2.3

H - hours; PEEP - positive end expiratory pressure. Results expressed as n (%), mean ± standard deviation or median (interquartile range).

### Respiratory features

**Figure 2:**
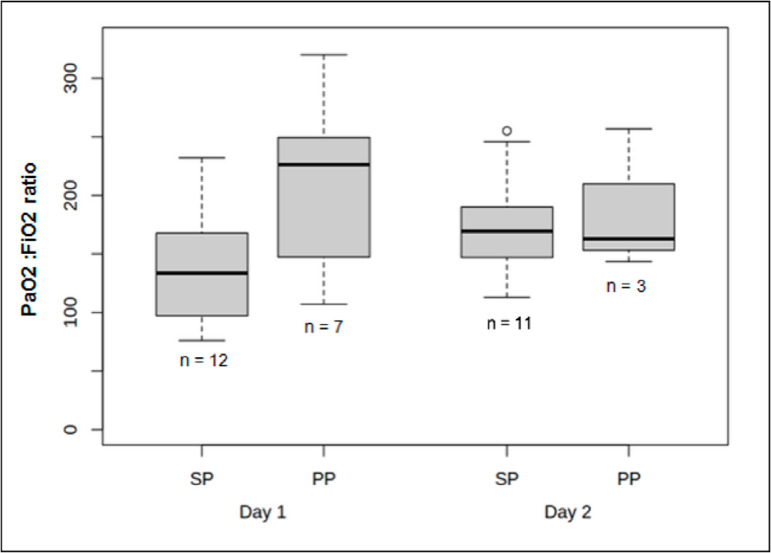
Partial pressure of arterial oxygen to fraction of inspired oxygen ratio evolution according to time and position.

All patients were intubated and mechanically ventilated with high FiO_2_ (mean 55.7% ± 17.6) and high PEEP (mean 12.7cmH_2_O ± 2). Tidal volume was targeted at 6mL per kilogram of predicted body weight, and PEEP level was titrated in accordance with the best respiratory-system static compliance. The median PaO_2_:FiO_2_ ratio in the supine position at H24 was 134 (interquartile range - IQR, 100 - 165). The median PaO_2_:FiO_2_ ratio at H48 was 169 (IQR, 148 - 186). In patients with a PaO_2_:FiO_2_ ratio lower than 150 after optimal treatment, prone positioning was performed for at least 16 consecutive hours. Seven patients needed prone positioning within the first 24 hours. The median PaO_2_:FiO_2_ ratio during the first session of the prone position was 226 (IQR, 147 - 250). Three patients needed a second prone position session within the first 48 hours. PaO_2_:FiO_2_ ratio evolution is represented in figure 2. Patients had a median compliance of 38mL/per cmH_2_O (IQR, 25 - 42) on day 1 and 40mL/per cmH_2_O (IQR, 30 - 44) on day 2.

PaO_2_ - partial pressure of oxygen; FiO_2_ - inspired oxygen ratio; SP - supine position; PP - prone position.

### Transpulmonary thermodilution features

The evolution of the main PiCCO™ parameters within the first 48 hours for every patient is reported in table 3. Upon admission, GEDV was normal (mean 738.8mL/m^2^ ± 209.2) and moderately increased at H48 (879mL/m^2^ ± 179). The cardiac index was subnormal upon admission (2.84L/minute/m^2^ ± 0.65) and at H48 (2.76L/minute/m^2^ ± 0.5). The systemic vascular resistance index was also normal (2386 dynes/second/cm^-5^/m^2^ ± 711). All patients presented an EVLW above 8mL/kg upon admission (17.9 ± 8.9), and 11/12 presented an EVLW above 11mL/kg. At H48, EVLW was slightly decreased (16.33 ± 4.1). Other PiCCO™ parameters were not systematically recorded and were excluded from the analysis.

**Table 3:** Main transpulmonary thermodilution parameters of the 12

	H0 (n = 12)	H24 (n = 9)	H48 (n = 9)
EVLW (mL/kg)	15 (13.5 - 18.7)	18 (13 - 21)	15 (13 - 21)
GEDV (mL/m^2^)	764 (582 - 861)	829 (737 - 1002)	884 (793 - 1003)
Cardiac index (L/min/m^2^)	2.7 (2.5 - 3)	2.8 (2.3 - 3.1)	2.76 (2.3 - 3.1)

H - hours; PEEP - positive end expiratory pressure. Results expressed as n (%), mean ± standard deviation or median (interquartile range).

### Other treatments

Patients were initially treated with antibiotics (cefotaxime and spiramycin). They were deeply sedated, and neuromuscular blockade was used for all patients. The depth of sedation was monitored by the bispectral index score (BIS), and neuromuscular blockade was monitored by train-of-four. Prophylactic anticoagulation was also administered. The fluid load between each transpulmonary thermodilution was limited (154mL ± 282). The maximal fluid load within 48 hours was 2,000mL for 1 patient, and three patients did not receive fluid load. Fluid loading was performed utilizing crystalloid fluid.

### Laboratory data

Table 4 shows some of the laboratory findings collected at H0 and at 48 hours. On admission, all patients presented with a biological inflammatory syndrome with high C-reactive protein (mean 197.3mmol/L ± 86.4) level and lymphopenia (0.7g/L ± 0.33), defined as a lymphocyte count lower than 1,500g/L. They did not present anemia (12.7g/dL ± 1.9) or thrombopenia (260g/L ± 68). The hematocrit level was similar at H0 (39.4% ± 5.3) and at H48 (37.2% ± 5.3), and the albumin level was moderately low on admission (24.4% ± 3.1). The serum lactate level was not elevated at H0 (1.6mmol/L ± 0.45) or at H48 (1.6 ± 0.4). Elevation of the serum urea (11.8mmol/L ± 8) and creatinine (121.4µmol/L ± 105.9) levels was variable among patients. No patients needed hemodialysis within the first 48 hours.

**Table 4:** Biological parameters of the 12 patients monitored with the PiCCO^™^ device

Analysis	H0 (n = 12)	H48 (n = 11)
Leucocyte count (g/L)	9.3 ± 3.2	9 ± 4.2
Neutrophil count (g/L)	8.1 ± 2.6	7.7 ± 3.9
Lymphocyte count (g/L)	0.7 ± 0.33	0.6 ± 0.2
Hemoglobin (g/dL)	12.7 ± 1.9	12 ± 2.2
Hematocrit (%)	39.4 ± 5.3	37.2 ± 5.3
Platelets (g/L)	260 ± 68	NA
Serum lactate (mmol/L)	1.6 ± 0.45	1.6 ± 0.4
Urea (mmol/L)	11.8 ± 8	NA
Creatinine (µmol/L)	121.4 ± 105.9	NA
Albumin (g/L)	24.4 ± 3.1	NA
C-reactive protein (mmol/L)	197.3 ± 86,4	NA

H - hours; NA - not analyzed. Results expressed as the mean ± standard deviation.

### Outcomes

All patients required mechanical ventilation. One patient died within the 24^th^ first hours because of the severity of respiratory failure. Eleven patients survived on day 28, and 10 (83%) survived on day 90. Seven patients (58%) underwent tracheotomy to aid weaning from mechanical ventilation. Four patients (33%) were discharged from the ICU on day 28, and 10 (83%) were discharged on day 90. Seven patients (58%) were discharged from the hospital on day 90.

## DISCUSSION

Among this cohort of 12 patients with severe COVID-19 pneumonia requiring mechanical ventilation, all of them presented pulmonary edema without evidence of cardiogenic origin or fluid overload.

### Characterization of pulmonary impairment

According to the Berlin definition, acute respiratory distress syndrome (ARDS) is a type of acute diffuse inflammatory lung injury that leads to increased pulmonary vascular permeability, increased lung weight, and loss of aerated lung tissue.^(10)^ Typical chest computed tomography (CT) scan findings (bilateral pulmonary parenchymal ground-glass, consolidative pulmonary opacities) are consistent with pulmonary edema.^(11)^ Diffuse alveolar damage, which is the morphological hallmark of ARDS, was found on a COVID-19 patient’s autopsy.^(12,13)^ In a four-patient autopsy series, Fox et al. described heavy lungs with diffuse edema consistent with the clinical diagnosis of ARDS.^(14)^ As presented in our results, static compliance of the respiratory system was moderately altered (median 38mL per cm of water). In a cohort of 66 intubated patients in Boston, Ziehr et al. showed similar results.^(15)^ Among these patients, 56 met the Berlin criteria for ARDS, and the mean static compliance of the respiratory system was 35mL per cmH_2_O (IQR, 30 - 43). Gattinoni et al. described a dissociation between hypoxemia severity and maintenance of relatively good respiratory mechanics with a median respiratory system compliance of approximately 50mL/cmH_2_O.^(16)^ They proposed a classification of two types of patients who could be distinguishable by chest CT scan.^(16)^ It is unclear whether these two presentations are distinct or are a kind of continuum mediated by potential self-inflicted lung injury.^(17)^ We did not perform a chest CT scan for all our patients because of the massive flood of critical patients.

### Respiratory care management

We chose to use high PEEP titrated in accordance with the best respiratory-system static compliance. In a recent study, Beloncle et al. suggested that a specific ratio could be used to identify highly recruitable patients from poorly recruitable patients.^(18)^ This strategy could be more precise to optimize the PEEP level. Prone positioning improves gas exchange in ARDS.^(19)^ Prone positioning was performed in six patients with a PaO_2_:FiO_2_ ratio less than 150 and improved their PaO_2_:FiO_2_ ratio. Another larger cohort showed the role of prone positioning in this situation.^(15,20)^ Respiratory support management was based on hypoxemia severity as defined by the PaO_2_:FiO_2_ ratio following French ARDS guidelines.^(21)^

### Transpulmonary thermodilution

We occasionally chose to use transpulmonary thermodilution to better understand this unknown pathology. To our knowledge, this is the first study that described early in vivo lung impairment using transpulmonary thermodilution in these patients. Among the 12 patients monitored by the PiCCO™ device, only one had EVLW < 12mL/kg upon admission. Hemodynamic features, low catecholamine doses and low lactate levels are not consistent with cardiogenic shock. Furthermore, 9 of the 12 patients underwent echocardiography. None had elevated left ventricular pressure assessed by E/E’. In mechanically ventilated ICU patients, E/E’ determination using tissue Doppler imaging closely approximates pulmonary artery occlusion pressure.^(22)^ None of the patients presented acute right ventricular failure. The inferior vena cava diameter was moderately increased, but patients underwent mechanical ventilation with high PEEP (mean 20.1 ± 2.4). The accuracy of the EVLW measurement by the PiCCO™ device is validated against the gold standard gravimetric measurement in animal and brain-dead patient models.^(23,24)^ It is now accepted that a normal EVLW value should approximate 7mL/kg and not exceed 10mL/kg (indexed by predicted body weight).^(25,26)^ Furthermore, a level of 14.6mL/kg could represent a 99% positive predictive value for diffuse alveolar damage.^(27)^ Elevated EVLW also appears to be a good predictor of mortality in critically ill patients.^(28)^ Tagami et al. suggested that delta-EVLW (the decrease in EVLW during the first 48 hours) could be associated with 28-day survival in ARDS.^(29)^

The presence of radiographic abnormalities and the severity of hypoxemia despite positive end expiratory pressure and hemodynamic features in these patients are compatible with ARDS according to the Berlin criteria.^(10)^

### Limitations

Our study has several limitations mostly related to its retrospective character, to the small number of patients and to the exceptional flood of patients inducing work overload. The small number of patients is related to the number of PiCCO™ monitors available and because we chose to use this technique upon admission. The different data were reported on the flowsheet of the patients. These data were reported by an experienced team, but only the main parameters were recorded. Unfortunately, the pulmonary vascular permeability index (PVPI) was not reported. Monnet et al. showed that PVPI allows hydrostatic pulmonary edema from increased permeability pulmonary edema differentiation, with a cutoff PVPI value of 3.^(30)^ This parameter could have allowed us to precisely determine the mechanism of pulmonary edema and could have provided an additional argument for ARDS. Moreover, one patient died within the first 24 hours, and transpulmonary thermodilution parameters were missing for 2 patients at H24 and 2 other patients at H48, which means that only parameters of nine patients are reported at H24 and H48. Because of the lack of several biological parameters in some patients at H48, we chose not to analyze these parameters. It is important to keep in mind that transpulmonary thermodilution has some limits.^(31)^ Extravascular lung water is unreliable in further situations, such as pulmonary embolism, lung resection and large pleural effusion. Based on radiographic data and past medical history, none of these patients had large pleural effusion or lung resection. As mentioned above, we did not perform chest CT scans, and we were not able to rule out pulmonary thromboembolism in these patients; however, we did not identify acute right ventricular failure on echocardiography. Therefore, these arguments support the reliability of EVLW in these patients. Global end diastolic volume indexed is less accurate than pulmonary artery occlusion pressure to diagnose left ventricular failure, but clinical and hemodynamic abnormalities and echocardiographic data were not compatible with a cardiogenic origin.

## CONCLUSION

This retrospective study specifies lung injury characteristics in severe COVID-19 pneumonia using transpulmonary thermodilution with a PiCCO™ device. These characteristics are consistent with pulmonary edema without evidence of a cardiogenic origin or fluid overload, favoring acute respiratory distress syndrome diagnosis. Respiratory management of these severe patients should probably follow acute respiratory distress syndrome guidelines. Further prospective studies are needed to specify respiratory impairment in patients with severe COVID-19 pneumonia and optimize respiratory care.
